# Models of care and the role of clinical pharmacists in UK primary care for older adults: A scoping review protocol

**DOI:** 10.1371/journal.pone.0276471

**Published:** 2023-07-27

**Authors:** Nathan Davies, Vladimir Kolodin, Abi Woodward, Cini Bhanu, Yogini Jani, Jill Manthorpe, Mine Orlu, Kritika Samsi, Alice Burnand, Victoria Vickerstaff, Emily West, Jane Wilcock, Greta Rait

**Affiliations:** 1 Research Department of Primary Care and Population Health, Centre for Ageing Population Studies, University College London, London, United Kingdom; 2 Research Department of Practice and Policy, School of Pharmacy, University College London, London, United Kingdom; 3 Centre for Medicines Optimisation Research and Education, University College London Hospitals NHS Foundation Trust, London, United Kingdom; 4 NIHR Applied Research Collaborative (ARC) South London, King’s College London, London, United Kingdom; 5 NIHR Policy Research Unit in Health & Social Care Workforce, King’s College London, London, United Kingdom; 6 Research Department of Pharmaceutics, UCL School of Pharmacy, University College London, London, United Kingdom; 7 Department of Clinical and Movement Neurosciences, University College London, London, United Kingdom; 8 Research Department of Primary Care and Population Health, PRIMENT Clinical Trials Unit, University College London, London, United Kingdom; Xiamen University - Malaysia Campus: Xiamen University - Malaysia, MALAYSIA

## Abstract

**Introduction:**

There has been global investment of new ways of working to support workforce pressures, including investment in clinical pharmacists working in primary care by the NHS in the England. Clinical pharmacists are well suited to support older adults who have multiple long-term conditions and are on multiple medications. It is important to establish an evidence base for the role of clinical pharmacists in supporting older adults in primary care, to inform strategic and research priorities. The aim of this scoping review is to identify, map and describe existing research and policy/guidance on the role of clinical pharmacists in primary care supporting older adults, and the models of care they provide.

**Methods and analysis:**

A scoping review guided by the Joanne Briggs Institute methodology for scoping reviews, using a three-step strategy. We will search Medline, CINAHL, Scopus, EMBASE, Web of Science, PSYCHInfo, and Cochrane for English language articles, from 2015 –present day. Grey literature will be searched using Grey Matters guidelines, the Index of Grey Literature and Alternative Sources and Resources, and Google keyword searching. References of all included sources will be hand searched to identify further resources. Using the Population, Concept and Context framework for inclusion and exclusion criteria, articles will be independently screened by two reviewers. The inclusion and exclusion criteria will be refined after we become familiar with the search results, following the iterative nature of a scoping review. Data will be extracted using a data extraction tool using Microsoft Excel and presented using a narrative synthesis approach.

**Ethics and dissemination:**

Ethical approval is not required for this review. Review findings will be disseminated in academic conferences and used to inform subsequent qualitative research. Findings will be published and shared with relevant local and national organisations.

## Introduction

General practice and primary care are undergoing a workforce crisis globally [1,2]. Growing demand, an ageing population, and more people living with complex or multiple long-term conditions, including dementia, have changed the landscape of primary care. Workforce challenges in general practice; specifically general practitioner (GP) recruitment and retention, are compounding problems [1,3]. To meet these challenges and provide high quality care, a diversity of professions in primary care is needed. There is currently a broadening of skills and specialisms in primary care teams internationally, with care being delivered by multi-disciplinary teams including GPs, specialist nurses, occupational therapists, paramedics, pharmacists and social prescribers, among others [4,5].

Since the 1980s, pharmacists have increasingly provided direct patient care, and this has been reported in many countries including the United States, Australia, New Zealand, Malaysia, the Netherlands and the UK for example [6–11]. Their work within primary care has evolved to be more clinically orientated. Clinical pharmacists may work with community pharmacists, who provide patients with their medications. However, a clinical pharmacist will also conduct clinical assessments and prescribe treatment, care management of patients with long-term diseases, and clinical medication reviews to proactively help people with complex polypharmacy [10,12–14]. Clinical pharmacists also have a strategic role in implementing national health priorities at local level, maximising benefit whilst minimising risk associated with medicines and designing treatment pathways for patients [14]. NHS England defines a clinical pharmacist as “*… highly qualified experts in medicines and can help people in a range of ways*. *This includes carrying out structured medication reviews for patients with ongoing health problems and improving patient safety*, *outcomes and value through a person-centred approach*.” [15].

In 2015 there was substantial investment in pharmacists in the UK. NHS England launched the pilot of the Clinical Pharmacists in General Practice scheme (CPGP) and its associated training programmes, as part of the NHS Five Year Forward View [16] and revised arrangements for general practice contracts [17]. Similar programmes were also launched in the devolved nations of the UK during this period [18,19].

This CPGP pilot involved 490 clinical pharmacists being embedded across 658 GP practices aiming to ease GP workload and improve patient access. An independent evaluation of the pilot concluded clinical pharmacists have made a substantial contribution to the primary care skill mix, including contributing to patient safety, healthy lifestyles, and medication knowledge across the primary care team [20]. Subsequently, the government made a commitment to an additional 7,500 clinical pharmacists in primary care networks by 2024 [21,22]. Following the CPGP pilot two cross-sectional surveys identified a variety of enhanced responsibilities of clinical pharmacists in primary care including: medicine reconciliation, telephone support for patients, and face to face medication review [23,24]. Other studies focused on workforce elements including integration within the primary care and practice environment [12,25]. Although there is evidence of the benefit of community pharmacy led services and pharmacists in hospital settings, there is a paucity of evidence within primary care, specifically related to clinical pharmacists [26,27].

Research on the benefits of having a clinical pharmacist in primary care for the general population have included identifying medicines-related problems, reduction in medication waste, and medicines optimisation [24,28]. Interviews with primary care teams and patients identified improved patient relationships, increased patient safety, enhanced cost savings and, importantly for patients, improved accessibility, with clinical pharmacists able to see patients much quicker than a GP or practice nurse [29–31].

The UK is seeing a rise in the ageing population, many of whom (67%) are living with two or more long term multiple conditions [32], including dementia and have associated high medication use and more rapid hospital discharge. Such factors have increased GPs’ workloads [33]. There is currently no cure for dementia, so the focus is on symptom management, providing good quality care, with an emphasis on living well and quality of life. Medicines play a key role in the management of symptoms [34], coupled with 91.8% of people living with dementia also having another health condition [35]. The presence of multiple long-term conditions leads to complex management and the use of complex drug regimes, with many people taking multiple medications (polypharmacy) which require ongoing management and review. Medication errors and overprescribing are global challenges [11,36,37], and has been highlighted as a serious problem in the UK with physical and mental impacts on patients, hospital visits and preventable admissions, premature deaths and increased costs for the NHS [28]. Overprescribing may also disproportionately affect Black, Asian and ethnic minority communities in the UK [28,38]. The role of the clinical pharmacist in primary care and general practice has valuable potential for the wellbeing of older people.

As the numbers of clinical pharmacists expand in primary care, it is important to establish an evidence base for the role of clinical pharmacists in supporting older adults in primary care including those living with dementia, to inform strategic and research priorities. Importantly this review will highlight gaps in workforce planning, strategy, commissioning, and research to inform future work and priorities in research, policy, and practice. We will identify different models of care, by which we mean what services are provided, what they do and who employs the pharmacists.

A preliminary search of Prospero, the Cochrane Database of Systematic Reviews and Joanna Briggs Institute (JBI) Evidence Synthesis was conducted in 2022 and no systematic reviews or scoping reviews on the topic were identified, either published or underway.

### Research questions

The overarching aim of this review is to identify, map and describe existing knowledge and research on the role of clinical pharmacists in primary care supporting older adults, and the models of care.

#### Primary review question

What research has been conducted on clinical pharmacy services used in primary care for older adults (over the age of 65 years) in the UK?

### Secondary review questions

- What is the role of clinical pharmacists, and what models of clinical pharmacy are used in primary care for older adults?
- What are professional perceptions of the role of clinical pharmacists who are involved in the care of older adults in primary care?
- What are the experiences of older adults and their carers (family and friends) who have encountered clinical pharmacists in primary care?
- What are the experiences of older adults from minority ethnic groups and those from social deprivation?
- What is the role of clinical pharmacists, and what models of clinical pharmacy are used in primary care for people with dementia?

## Methods

### Design

The proposed scoping review will be conducted in accordance with the JBI methodology for scoping reviews [39,40]. The review will be guided by the PCC (Participant, concept, and context) framework as recommended in the JBI scoping review methodology, to inform our review questions, search strategy, and inclusion/exclusion criteria. This protocol is reported using the Preferred Reporting Items for Systematic Review and Meta-Analysis extension for scoping reviews (PRISMA-ScR) [41] and Preferred Reporting Items for Systematic Review and Meta-Analysis Protocols (PRISMA-P) checklist for systematic review protocols (see S1 Appendix) [42] adapted using guidance by JBI for best practice reporting of scoping review protocols [43].

### Eligibility criteria

An overview of eligibility criteria is provided in Table 1.

**Table 1:** Overview of inclusion and exclusion criteria.

	*Included*	*Excluded*
***Population***	Older people (65 years and above) or if the paper describes them as older adults/people	Mixed population with no separate analysis on older people
** **	Professionals caring for older adults	Those under 65 years
** **	Family and friends who are carers of older adults	
***Concept***	Clinical pharmacist role	Community pharmacy
	Clinical pharmacy in primary care	
** **	Focus on what clinical pharmacy services are delivered and how	
** **	Experiences of services from professionals, family carers and older adults’ perspectives	
***Context***	Primary care	Studies outside the UK
	UK	Studies prior to 2015
	Published since 2015 and the introduction of the clinical pharmacist in general practice	Studies of the community pharmacy role
	Care homes	Dentistry and optometry services
		Non-English language

#### Participants

The population for this review is older people in primary care. In the UK the definition of an older adult is generally someone over the age of 65 years [44], and is the definition adopted for this review. We will also include papers which do not specify the age of participants but refer to them as ‘older’. Sources which do not specify their focus is on older adults, or those over 65 years will not be included. Sources which have a mix of participants’ ages will be included if they have a separate discussion or analysis on older adults. Sources which include professionals, older adults and carers will be included in the review. The definition of professionals will include doctors, pharmacists, practice nurses and other staff in primary care. The review will focus on unpaid carers who are a friend or family member of an older person who regularly takes care of the person due to their illness, frailty, or disability. Carers will be included with no restriction on age or other demographics.

#### Concept

Studies and resources that relate to clinical pharmacists who work in primary care will be included. Our definition of primary care is provided below in context.

We will include resources, including research, policy and grey literature, reporting: what services and how services are delivered, who is using services, the frequency with which services are being used, whether services are being responded to positively or otherwise. There are also likely to be resources on professionals’ experiences of these services–both from the primary care and pharmacy perspective. Similarly, data may include patient and carer experiences in using (or not using) such services as well as resources, publications and reviews that explore the role of clinical pharmacists in primary care. In initial screening we will include condition specific sources (e.g. studies which focus on older adults with COPD), however we will review this decision in light of the results of abstract screening and refine our criteria as appropriate at that point. This is in line with the iterative nature of a scoping review (40).

#### Context

The context for this review is primary care, which is defined for this review as services which provide the first point of contact in the healthcare system [45], this includes but is not limited to general practice. For the purposes of this review, we will not include dental, optometry or community pharmacy services. However, we are aware clinical pharmacists may work in community teams and support community pharmacy services as part of their role employed by another primary care organisation (i.e. GP practice, primary care network, integrated care systems) and sources focussing on clinical pharmacists from those organisations will be included. We will include care delivered by clinical pharmacists to care homes. Resources and publications for this review will be from the UK context to account for specificities in service provision and the role of both primary care and pharmacy.

#### Types of sources

This scoping review will consider all primary research studies (qualitative and quantitative), systematic reviews, meta-analyses, letters to editors, commentaries, blogs and grey literature.

We will actively search for and include grey literature such as: theses, dissertations, trade publications, national policy and guidelines, reports, websites, conference posters, preprints, and others. This is relevant as this is a new emerging field, and more information may be published in these sources. This will minimise publication bias and maximise our understanding of the landscape. Non-English language studies and those published prior to 2015 will be excluded. We selected 2015 as the cut-off date for three reasons: 1) this was the start of the pilot of clinical pharmacists working in general practice and primary care in England [20]; 2) the timepoint of investment by the Scottish Government to support recruiting additional pharmacists to support the care of patients with long term conditions, freeing up GP time [18] and finally 3) the period when there significant investment in recruitment of clinical pharmacist in primary care teams in Wales [19]. However, we acknowledge that some parts of the UK may have other models of care which pre-date our cut-off date and this will be acknowledged in our review.

### Search strategy

The JBI recommends a three-step search strategy [40], firstly an initial limited search of two databases will be undertaken (CINAHL and MedLine) based on initial search terms developed from existing literature and discussions with an information specialist from the University library. We will use a combination of keywords and Medical Subject Headings (MeSH) terms which will be adapted for the various databases, combined using Boolean operators (‘AND’ ‘OR’). Results will be screened at title and abstract level to ensure sensitivity and specificity of the search strategy, ensuring that key articles already known to the research team are captured in the results. The search strategy will then be refined using search terms from identified articles in step one. For step two the revised search will be re-run in the initial two databases, as well as, Scopus, EMBASE, Web of Science, PSYCHInfo, and Cochrane (see S1 Appendix for example full search strategy). Grey literature will be searched guided by Grey Matters guidelines, the Index of Grey Literature and Alternative Sources and Resources and Google keyword searching. Following this, the final step will include hand searching the reference lists of included articles, contacting authors for further information if necessary and to identify any further sources, and finally we will conduct citation tracking using Google Scholar. We will work with our study patient and public involvement group (PPI) as well as key stakeholders to identify any additional sources our searches may not have identified.

Search terms will be identified prior to starting the review, however as the JBI Manual states the search for a scoping review may be iterative as reviewers become more familiar with the evidence base [40], we will therefore revise our search strategy as needed as we proceed with the review.

### Study/Source of evidence selection

Following the search, all identified citations will be collated and uploaded into Endnote, and duplicates removed. References will be imported into Rayann for screening [46]. Following a pilot test and initial screening to refine the search, titles and abstracts will then be screened by one reviewer (VK, AB, DL or ND) for assessment against the inclusion criteria for the review and a random 10% reviewed by a second reviewer (VK, AB, DL or ND). Potentially relevant sources will be retrieved in full. The full text of selected citations will be assessed in detail against the inclusion criteria by one reviewer (VK, AB, or ND) and 10% reviewed by a second reviewer (VK, AB, DL or ND). Reasons for exclusion of sources of evidence at full text that do not meet the inclusion criteria will be recorded and reported in the scoping review. Any disagreements that arise between the reviewers at each stage of the selection process will be resolved through discussion, or with an additional reviewer/s. The results of the search and the study inclusion process will be reported in full in the final scoping review and presented in a Preferred Reporting Items for Systematic Reviews and Meta-analyses extension for scoping review (PRISMA-ScR) flow diagram [41]. We will continually review our eligibility criteria and focus of the review based on the identified sources, and revise the eligibility criteria as needed in line with the iterative nature of scoping reviews [40]. We will not conduct quality assessment of included articles as this is not recommended by scoping review guidance [39,47].

### Data extraction

The data from the included sources will be extracted using a data extraction tool developed by the research team using Microsoft Excel. The tool will be tested on two sources initially to ensure all relevant information is being collected and modified as required. This will be conducted by two independent reviewers (VK or AB) and individual extraction compared, any disagreements that arise between the reviewers will be resolved through discussion, or with an additional reviewer/s. Following this the remaining included sources will be extracted by two independent reviewers (VK,AB, or AW). Throughout the review process the extraction tool will be modified as necessary as we learn more about the field and results, this will be detailed in the review. If appropriate, authors of papers will be contacted to request missing or additional data, where required. The following information will be extracted:

- Author(s)
- Year of publication
- Country of origin
- Source type (i.e. journal article, report, website)
- Aims
- Study design (if applicable)
- Participant demographics
- Model of clinical pharmacy (i.e. services delivered, who employs the pharmacist, what they do)
- Clinical pharmacy role/definition
- Key findings qualitative
- Key findings quantitative
- Conclusions
- Strengths and limitations
- Implications for future research
- Implications for policy and practice
- Role of communication
- Context and special requirements when work with minority ethnic groups

### Data analysis and presentation

Findings will be presented separately for each of the research questions above. Initially, providing a taxonomy or figure to reflect the breadth and areas of research in this field. This will be followed by a series of tables and narrative summary of the included sources. Although scoping review guidance does not recommend the use of thematic analysis to analyse the data we will use the principles of codebook thematic analysis [48] to identify key themes across the sources and enable us to narratively present the remaining findings from the sources included. We will highlight gaps in the literature and identify future priorities for research, policy, and practice. The report will follow the PRISMA guidance for scoping reviews [41].

### PPI and stakeholder engagement

Our PPI group consisting of two family carers of older adults has been embedded in our research from the outset, advising on resources to consider and sources of information. We will present our emerging findings throughout the process to our PPI group and additional stakeholders including older people, family and friends who are carers, and professionals from within primary care, to inform our synthesis and identify gaps in the literature. During initial consolations, PPI representative suggested to focus on the role of communication between clinical pharmacist and patients, as well as specific approaches when working with people from minority ethnic groups.

### Impact

This scoping review will increase the understanding of the landscape of clinical pharmacy in primary care, specifically in relation to older adults. The information from the scoping review together with primary research to be conducted alongside the review will be presented in a series of stakeholder engagement workshops to produce a list of priorities for research. The outcome of this literature review will identify key services being delivered, patterns of engagement and utilisation of services, and perceptions of key stakeholders regarding these services. The review will provide evidence for clinical pharmacy services and older adults in primary care, directly influencing new and emerging agendas of the primary care workforce and the role of clinical pharmacists in the UK.

## Supporting information

S1 Checklist PRISMA-P 2015 checklist. (DOCX)

S1 Appendix Search strategy for MEDLINE. (DOCX)

## References

[1] Marshall M, Ikpoh M. The workforce crisis in general practice. British Journal of General Practice; 2022. p. 204–5. doi: 10.3399/bjgp22X719213 35483948 PMC11189033

[2] Papp M, Kőrösi L, Sándor J, Nagy C, Juhász A, Ádány R. Workforce crisis in primary healthcare worldwide: Hungarian example in a longitudinal follow-up study. BMJ open. 2019;9(7):e024957. doi: 10.1136/bmjopen-2018-024957 31340955 PMC6661691

[3] Baird B, Charles A, Honeyman M, Maguire D, Das P. Understanding pressures in general practice. 2016 [Available from: https://www.kingsfund.org.uk/sites/default/files/field/field_publication_file/Understanding-GP-pressures-Kings-Fund-May-2016.pdf.

[4] Royal College of General Practitioners. Fit for the future: a vision for general practice. London; 2019.

[5] Freund T, Everett C, Griffiths P, Hudon C, Naccarella L, Laurant M. Skill mix, roles and remuneration in the primary care workforce: who are the healthcare professionals in the primary care teams across the world? International journal of nursing studies. 2015;52(3):727–43. doi: 10.1016/j.ijnurstu.2014.11.014 25577306

[6] Rueben A, Forsyth P, Thomson AH. Professional development beyond foundation training: a study of pharmacists working in Scotland. International Journal of Pharmacy Practice. 2020;28(2):165–72. doi: 10.1111/ijpp.12585 31583789

[7] Samir Abdin M, Grenier-Gosselin L, Guénette L. Impact of pharmacists’ interventions on the pharmacotherapy of patients with complex needs monitored in multidisciplinary primary care teams. International Journal of Pharmacy Practice. 2020;28(1):75–83. doi: 10.1111/ijpp.12577 31468599

[8] Moreno G, Lonowski S, Fu J, Chon JS, Whitmire N, Vasquez C, et al. Physician experiences with clinical pharmacists in primary care teams. Journal of the American Pharmacists Association. 2017;57(6):686–91. doi: 10.1016/j.japh.2017.06.018 28811089

[9] San Saw P, Nissen LM, Freeman C, Wong PS, Mak V. Health care consumers’ perspectives on pharmacist integration into private general practitioner clinics in Malaysia: a qualitative study. Patient preference and adherence. 2015;9:467. doi: 10.2147/PPA.S73953 25834411 PMC4372008

[10] Baker S, Lee YP, Hattingh HL. An evaluation of the role of practice pharmacists in Australia: a mixed methods study. International journal of clinical pharmacy. 2019;41(2):504–15. 30879214 10.1007/s11096-019-00807-5

[11] Haua R, Harrison J, Aspden T. Pharmacist integration into general practice in New Zealand. Journal of Primary Health Care. 2019;11(2):159–69. doi: 10.1071/HC18103 32171359

[12] Bradley F, Seston E, Mannall C, Cutts C. Evolution of the general practice pharmacist’s role in England: a longitudinal study. British Journal of General Practice. 2018;68(675):e727–e34. doi: 10.3399/bjgp18X698849 30154077 PMC6146003

[13] Khaira M, Mathers A, Benny Gerard N, Dolovich L. The evolving role and impact of integrating pharmacists into primary care teams: Experience from Ontario, Canada. Pharmacy. 2020;8(4):234. doi: 10.3390/pharmacy8040234 33297509 PMC7768418

[14] Royal Pharmaceutical Society. Primary Care Pharmacy https://www.rpharms.com/resources/careers-information/career-options-in-pharmacy/primary-care-pharmacy.

[15] NHS England. Clinical pharmacists [Available from: https://www.england.nhs.uk/gp/expanding-our-workforce/cp-gp/.

[16] NHS. Five year forward view 2014 [Available from: https://www.england.nhs.uk/wp-content/uploads/2014/10/5yfv-web.pdf.

[17] Royal College of General Practitioners, British Medical Association, NHS England. Building the workforce—the new deal for general practice 2015 [Available from: https://www.england.nhs.uk/commissioning/wp-content/uploads/sites/12/2015/01/building-the-workforce-new-deal-gp.pdf.

[18] Scottish Government. Primary care investment: three-year funding to support front-line community care 2015 [Available from: https://www.webarchive.org.uk/wayback/archive/20180529225529/https://news.gov.scot/news/primary-care-investment.

[19] Welsh Government. Clinical pharmacists in GP practices 2016 [Available from: http://www.wales.nhs.uk/news/40188.

[20] Mann C, Anderson C, Avery AJ, Waring J, Boyd M. Clinical Pharmacists In General Practice: Pilot Scheme: Independent Evaluation Report: Full Report. 2018.

[21] NHS England. The NHS Long Term Plan. London; 2019.

[22] NHS England BMA. Investment and evolution: a five-year framework for GP contract reform to implement The NHS Long Term Plan. 2019 [Available from: https://www.england.nhs.uk/wp-content/uploads/2019/01/gp-contract-2019.pdf.

[23] Alshehri AA, Cheema E, Yahyouche A, Haque MS, Jalal Z. Evaluating the role and integration of general practice pharmacists in England: a cross-sectional study. International Journal of Clinical Pharmacy. 2021:1–10. 34080088 10.1007/s11096-021-01291-6 PMC8642258

[24] Savickas V, Foreman E, Ladva A, Bhamra SK, Sharma R, Corlett SA. Pharmacy services and role development in UK general practice: a cross-sectional survey. International Journal of Pharmacy Practice. 2020;29(1):37–44. 10.1111/ijpp.12653 32627272

[25] Stewart D, Maclure K, Newham R, Gibson-Smith K, Bruce R, Cunningham S, et al. A cross-sectional survey of the pharmacy workforce in general practice in Scotland. Family Practice. 2019;37(2):206–12. 10.1093/fampra/cmz052 31536620

[26] Weir NM, Preston K, Newham R, Bennie M. Development of a primary care pharmacy outcomes framework: An umbrella literature review. Research in Social and Administrative Pharmacy. 2021. doi: 10.1016/j.sapharm.2021.07.010 34353755

[27] Anderson C, Zhan K, Boyd M, Mann C. The role of pharmacists in general practice: a realist review. Research in Social and Administrative Pharmacy. 2019;15(4):338–45. doi: 10.1016/j.sapharm.2018.06.001 29907317

[28] Ridge K. Good for you, good for us, good for everybody A plan to reduce overprescribing to make patient care better and safer, support the NHS, and reduce carbon emissions. London; 2021.

[29] Ryan K, Patel N, Lau WM, Abu-Elmagd H, Stretch G, Pinney H. Pharmacists in general practice: a qualitative interview case study of stakeholders’ experiences in a West London GP federation. BMC Health Services Research. 2018;18(1):234. doi: 10.1186/s12913-018-3056-3 29609603 PMC5879559

[30] Karampatakis GD, Patel N, Stretch G, Ryan K. Patients’ experiences of pharmacists in general practice: an exploratory qualitative study. BMC Family Practice. 2021;22(1):48. doi: 10.1186/s12875-021-01393-0 33673805 PMC7935482

[31] Hampson N, Ruane S. The value of pharmacists in general practice: perspectives of general practitioners—an exploratory interview study. International Journal of Clinical Pharmacy. 2019;41(2):496–503. doi: 10.1007/s11096-019-00795-6 30864082

[32] Salive ME. Multimorbidity in older adults. Epidemiologic reviews. 2013;35(1):75–83. doi: 10.1093/epirev/mxs009 23372025

[33] Hajat C, Stein E. The global burden of multiple chronic conditions: a narrative review. Preventive medicine reports. 2018;12:284–93. doi: 10.1016/j.pmedr.2018.10.008 30406006 PMC6214883

[34] Donegan K, Fox N, Black N, Livingston G, Banerjee S, Burns A. Trends in diagnosis and treatment for people with dementia in the UK from 2005 to 2015: a longitudinal retrospective cohort study. The Lancet Public Health. 2017;2(3):e149–e56. doi: 10.1016/S2468-2667(17)30031-2 29253388

[35] Browne J, Edwards DA, Rhodes KM, Brimicombe DJ, Payne RA. Association of comorbidity and health service usage among patients with dementia in the UK: a population-based study. BMJ open. 2017;7(3):e012546. doi: 10.1136/bmjopen-2016-012546 28279992 PMC5353300

[36] Claesson CB, Burman K, Nilsson JLG, Vinge E. Prescription errors detected by Swedish pharmacists. International Journal of Pharmacy Practice. 1995;3(3):151–6.

[37] Avery AJ, Ghaleb M, Barber N, Franklin BD, Armstrong SJ, Serumaga B, et al. The prevalence and nature of prescribing and monitoring errors in English general practice: a retrospective case note review. British Journal of General Practice. 2013;63(613):e543–e53. doi: 10.3399/bjgp13X670679 23972195 PMC3722831

[38] Secchi A, Booth A, Maidment I, Sud D, Zaman H. Medication management in M inority, A sian and B lack ethnic older people in the United Kingdom: A mixed‐studies systematic review. Journal of Clinical Pharmacy and Therapeutics. 2022. 10.1111/jcpt.13735 35844186

[39] Peters MDJ, Marnie C, Tricco AC, Pollock D, Munn Z, Alexander L, et al. Updated methodological guidance for the conduct of scoping reviews. JBI Evidence Synthesis. 2020;18(10). doi: 10.11124/JBIES-20-00167 33038124

[40] Peters MDJ, Godfrey C, McInerney P, Munn Z, Tricco AC, Khalil H. Chapter 11: Scoping Reviews. In: Aromataris E, (Editors). Mz, editors. JBI manual for evidence synthesis 2020.

[41] Tricco AC, Lillie E, Zarin W, O’Brien KK, Colquhoun H, Levac D, et al. PRISMA extension for scoping reviews (PRISMA-ScR): checklist and explanation. Annals of internal medicine. 2018;169(7):467–73. doi: 10.7326/M18-0850 30178033

[42] Shamseer L, Moher D, Clarke M, Ghersi D, Liberati A, Petticrew M, et al. Preferred reporting items for systematic review and meta-analysis protocols (PRISMA-P) 2015: elaboration and explanation. Bmj. 2015;349. doi: 10.1136/bmj.g7647 25555855

[43] Peters MDJ, Godfrey C, McInerney P, Khalil H, Larsen P, Marnie C, et al. Best practice guidance and reporting items for the development of scoping review protocols. JBI Evidence Synthesis. 2022;20(4). doi: 10.11124/JBIES-21-00242 35102103

[44] Age UK. Later life in the United Kingdom. 2019.

[45] NHS England. Primary Care Services [Available from: https://www.england.nhs.uk/get-involved/get-involved/how/primarycare/#:~:text=Primary%20care%20services%20provide%20the,optometry%20(eye%20health)%20services.

[46] Ouzzani M, Hammady H, Fedorowicz Z, Elmagarmid A. Rayyan—a web and mobile app for systematic reviews. Systematic reviews. 2016;5(1):1–10. doi: 10.1186/s13643-016-0384-4 27919275 PMC5139140

[47] Munn Z, Peters MDJ, Stern C, Tufanaru C, McArthur A, Aromataris E. Systematic review or scoping review? Guidance for authors when choosing between a systematic or scoping review approach. BMC Medical Research Methodology. 2018;18(1):143. doi: 10.1186/s12874-018-0611-x 30453902 PMC6245623

[48] Braun V, Clarke V. Thematic analysis: A practical guide: Sage; 2021.

