# Supplementary material for: Models of care and the role of clinical pharmacists in UK primary care for older adults: A scoping review protocol
Source: PLoS One. 2023 Jul 27;18(7):e0276471. doi: 10.1371/journal.pone.0276471 (PMC10374084; doi:10.1371/journal.pone.0276471)
Supplement: S1 Appendix — (DOCX) [file pone.0276471.s002.docx]

**S1 Appendix**

| # | Query |
| --- | --- |
| 1 | (primary care or primary health care or primary healthcare or primary care provider* or primary care facilit* or general practice or general practitioner* or GP).mp. [mp=title, book title, abstract, original title, name of substance word, subject heading word, floating sub-heading word, keyword heading word, organism supplementary concept word, protocol supplementary concept word, rare disease supplementary concept word, unique identifier, synonyms] |
| 2 | exp Primary Health Care/ |
| 3 | exp General Practice/ |
| 4 | 1 or 2 or 3 |
| 5 | (clinical pharmacist* or pharmacist*).mp. [mp=title, book title, abstract, original title, name of substance word, subject heading word, floating sub-heading word, keyword heading word, organism supplementary concept word, protocol supplementary concept word, rare disease supplementary concept word, unique identifier, synonyms] |
| 6 | exp Pharmacists/ |
| 7 | 5 or 6 |
| 8 | Age Factors/ |
| 9 | (age 65 and over).mp. [mp=title, book title, abstract, original title, name of substance word, subject heading word, floating sub-heading word, keyword heading word, organism supplementary concept word, protocol supplementary concept word, rare disease supplementary concept word, unique identifier, synonyms] |
| 10 | (age* or elderly or frailty or older adult*).mp. [mp=title, book title, abstract, original title, name of substance word, subject heading word, floating sub-heading word, keyword heading word, organism supplementary concept word, protocol supplementary concept word, rare disease supplementary concept word, unique identifier, synonyms] |
| 11 | 8 or 9 or 10 |
| 12 | 4 and 7 and 11 |
| 13 | limit 12 to (english language and yr="2015 -Current") |
| 14 | limit 13 to humans |
