# Supplementary material for: Models of care and the role of clinical pharmacists in UK primary care for older adults: A scoping review protocol
Source: PLoS One. 2023 Jul 27;18(7):e0276471. doi: 10.1371/journal.pone.0276471 (PMC10374084; doi:10.1371/journal.pone.0276471)
Supplement: Attachment — Submitted filename: Response to reviewers .pdf [file pone.0276471.s004.pdf]

UNIVERSITY COLLEGE LONDON
UCL Research Department of Primary care & Population Health

Centre for Ageing Population Studies

UCL Research Department of Primary Care & Population Health
Royal Free Hospital
Rowland Hill Street
London
NW3 2PF

03 April 2023

Dear Dr Surangi Jayakody,

We would like to thank both reviewers for their positive and helpful comments which we feel have strengthened our manuscript. All page numbers and line numbers refer to the tracked changes document.

Reviewer #1: There are few concerns which need to be cleared before publications and they are given as the comments.

Interesting and timely topic to explore.
Thank you we agree this is a very timely topic.

It would be better if the pre-determined tool can be mentioned.
It is not a tool which already exists but one which we will develop as a team – we have clarified this in the abstract and then on page 13 in the data extraction section we believe this is clearer. On page 13-14 we specify what is included in the data extraction tool/template.

Is this "older adults"? (page 7 line 174)
Yes, we have added older to this question.

It is better to add in the introduction why dementia is taken out of all the multi morbidities in elders.
We have added information on dementia on page 6 lines 131-136 and 149 to introduce this before the aims.

Why are the unpaid carers are excluded?
Unpaid carers (i.e. family and friends) are included – this is discussed on page 8 line 206-208 and again in the eligibility criteria table on page 11.

Are these consensus documents? (Page 10, line 239)
No we do not think these are consensus documents.

what about paid carers? (inclusion criteria)
We are including professionals caring for older adults, this is in our eligibility criteria table on page 11.

Reviewer #2: Thank you for the opportunity to review this interesting paper. I think that with the following amendments, this paper would be suitable for publication in PLOS ONE.

Abstract

Methods: the date (month/year- from 2015) the search will be conducted and any search limits (e.g. language- Only English language articles) need to be added.
We have added 2015-present day as it is not possible to specify a date to confirm when the final search will be completed as we would update this before publication of the finalised review. However, we will specify this in the main article when publishing. We have added the limit English language.

Methods
Data Extraction

Page 13- Following this the remaining included sources will be extracted by one reviewer (VK or AB).

Data need to be extracted from papers included in the scoping review by two or more independent reviewers using a data extraction tool.

Thank you for raising this we have amended the protocol see page 13 line 313.

We look forward to hearing your response to our revisions.

Yours sincerely,

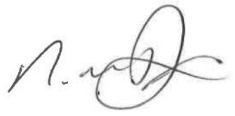

Dr Nathan Davies (on behalf of the authors)

Associate Professor of Ageing and Applied Health Research
